# Robust Correlation Analyses: False Positive and Power Validation Using a New Open Source Matlab Toolbox

**DOI:** 10.3389/fpsyg.2012.00606

**Published:** 2013-01-10

**Authors:** Cyril R. Pernet, Rand Wilcox, Guillaume A. Rousselet

**Affiliations:** ^1^Brain Research Imaging Center, Division of Clinical Neurosciences, University of EdinburghEdinburgh, UK; ^2^Department of Psychology, University of Southern CaliforniaLos Angeles, CA, USA; ^3^Centre for Cognitive Neuroimaging, Institute of Neuroscience and Psychology, College of Medical, Veterinary and Life Sciences, University of GlasgowGlasgow, UK

**Keywords:** robust statistics, correlation, power, outliers, MATLAB

## Abstract

Pearson’s correlation measures the strength of the association between two variables. The technique is, however, restricted to linear associations and is overly sensitive to outliers. Indeed, a single outlier can result in a highly inaccurate summary of the data. Yet, it remains the most commonly used measure of association in psychology research. Here we describe a free Matlab^(R)^ based toolbox (http://sourceforge.net/projects/robustcorrtool/) that computes robust measures of association between two or more random variables: the percentage-bend correlation and skipped-correlations. After illustrating how to use the toolbox, we show that robust methods, where outliers are down weighted or removed and accounted for in significance testing, provide better estimates of the true association with accurate false positive control and without loss of power. The different correlation methods were tested with normal data and normal data contaminated with marginal or bivariate outliers. We report estimates of effect size, false positive rate and power, and advise on which technique to use depending on the data at hand.

## Introduction

Robust statistical procedures have been developed since the 1960s (Tukey, 1960; Huber, 1964) to solve problems inherent in using classic parametric methods when assumptions are violated (Erceg-Hurn and Mirosevich, 2008). Although many scientists are aware of these techniques, and of their superiority in many cases, robust statistics are not widely used or even part of the standard curriculum. There are two reasons for this. First, no single method is optimal in all situations. Although least squares is a technique easy to compute in many situations, it is often disastrous and inappropriate (Wilcox, 2001) because assumptions are often not met (e.g., Micceri, 1989); leaving us to have to choose among multiple robust alternatives. Second, developers of statistical methods tend to provide code that is not sufficiently user-friendly. As a consequence, robust techniques remain underused and do not find their way into commercial software packages (Stromberg, 2004). Here, we present a free Matlab toolbox to perform robust correlation analyses (http://sourceforge.net/projects/robustcorrtool/). The toolbox contains several correlation techniques described in Wilcox (2012a). These techniques can also be found in separate R functions (R Development Core Team, 2011). In addition, the toolbox provides graphical outputs and tests of assumptions.

Generally, a correlation refers to any of a broad class of statistical relationships involving dependence. Correlation also refers to a broad class of statistical measures aimed at characterizing the strength of the association between two variables. Among these latter measures, Pearson’s correlation is the most widely used technique, despite its lack of robustness (Wilcox, 2012a,b). Indeed, Pearson’s correlation is overly sensitive to outliers; it is also affected by the magnitude of the slope around which points are clustered, by curvature, by the magnitude of the residuals, by the restriction of range, and by heteroscedasticity. Our toolbox computes robust alternatives: the percentage-bend correlation (Wilcox, 1994) and skipped-correlations (Wilcox, 2004). These alternatives have a practical value relative to the standard Pearson’s correlation because they estimate linear relationships and often provide better estimates of the true relationship between variables (Rousselet and Pernet, 2012). The percentage-bend correlation protects against marginal outliers without taking into account the overall structure of the data. Importantly, it estimates linear associations, but does not estimate Pearson’s: the results are not comparable across the [−1, +1] range. Skipped-correlations protect against bivariate outliers by taking into account the overall structure of the data, and Pearson’s skipped correlation is a direct reflection of Pearson’s *r*.

### Toolbox features

**Figure 1:**
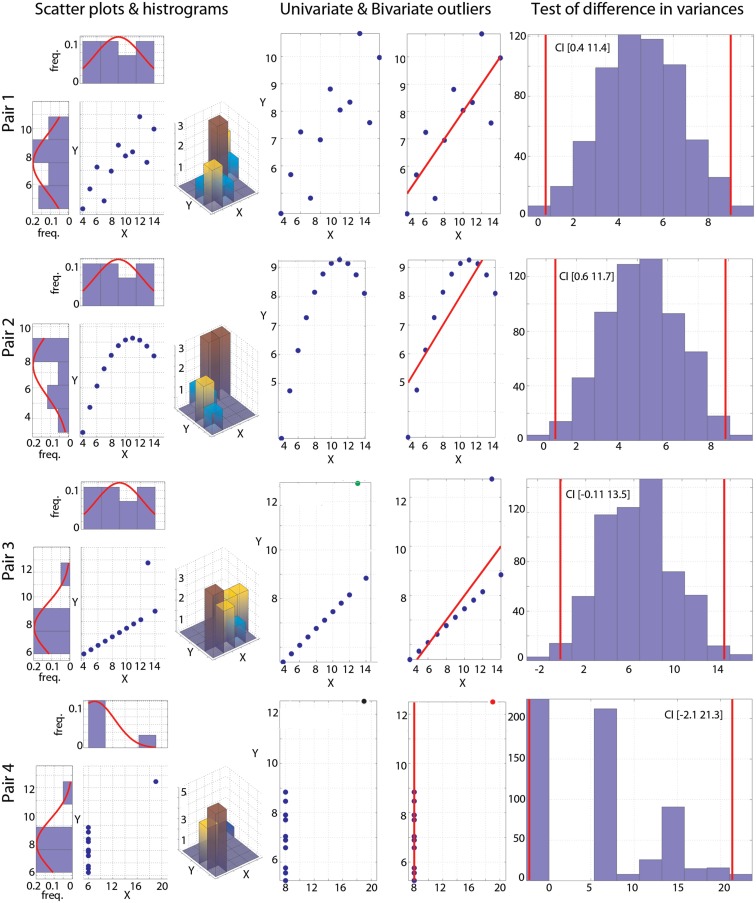
**Visualization of the Anscombe’s quartet**. Each pair is illustrated by a scatter plot and with univariate and bivariate histograms (left column). Outliers detected using the box-plot rule are plotted in the two middle columns: column 2 shows univariate outliers in *Y* (green) or in *X* and *Y* (black); column 3 shows bivariate outliers (red), with the best line fitted to the remaining points. Histograms (right column) show the bootstrapped variance differences. Vertical red lines indicate 95% CIs.

Alongside the computations of correlations, the toolbox includes tools for visualization and basic assumption checking. The *corr_normplot.m* function provides, in one figure, a scatterplot of the data, the marginal (normalized) histograms with the matching Gaussian curves, and the bivariate histogram (Figure 1, left column). The *joint_density.m* function plots both a mesh of the joint density and its isocontour. Although the joint density is similar to the bivariate histogram, it provides a better visualization of the bivariate space when there are many observations. Visualization is indeed the first step before computing any correlation: in some extreme situations, as in the case of split data clouds, a correlation analysis would be worthless (see e.g., Figure 1E in Rousselet and Pernet, 2012). Tests of correlations are sensitive to different features of the data. For instance, Pearson’s correlation is only meaningful for linear parametric models estimated via least squares, whilst Spearman’s correlation deals with monotonic associations in a more flexible manner. Both tests are however sensitive to heteroscedasticity (Wilcox and Muska, 2001). The toolbox thus provides tools to compute conditional means and variances (*conditional.m*) and to test variance homogeneity based on a percentile bootstrap with adjustment for small samples (*variance_homogeneity.m*). The function outputs the 95% confidence intervals (abbreviated CIs in the rest of the paper) and the histogram of the bootstrapped estimates (Figure 1, right column). In addition, because skewness can cause large deviation in correlation estimates, we included the Henze–Zirkler test for multivariate normality (*HZmvntest.m*). This function was implemented by Trujillo-Ortiz et al. (2007) and is distributed under DSB license with the toolbox. Finally, univariate and bivariate outlier detection can be performed using several techniques: box-plot rule, MAD-median rule, S-outliers (*detect_outliers.m* – Figure 1, middle columns; Appendix). The toolbox also computes Pearson’s (*Pearson.m*), Spearman’s (*Spearman.m*), percentage-bend (*bendcorr.m* – Wilcox, 1994), and skipped-correlations (*skipped_correlation.m* – Wilcox, 2004) with their 95% percentile bootstrap CIs.

## Methods

### Correlation measures

**Figure 2:**
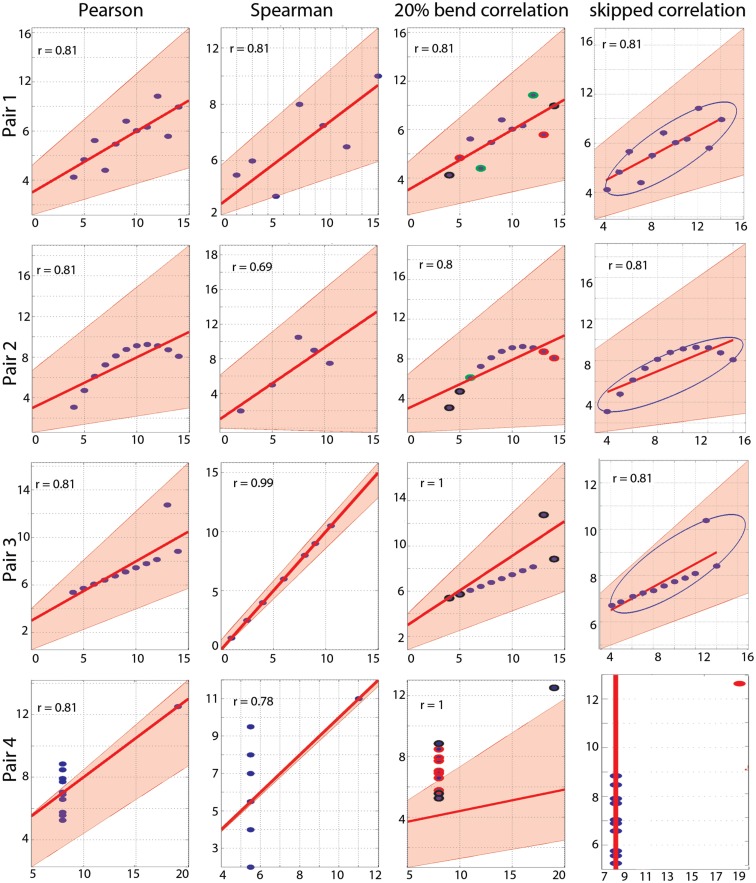
**Correlation results**. From left to right are illustrated Pearson’s, Spearman’s, 20% bend, and Pearson’s skipped-correlations with the 95% bootstrapped CIs as pink shaded areas. The scale for Spearman’s correlations differs from the others because ranked data are plotted. For the 20% bend correlation, red indicates data bent in *X*, green in *Y* and black in both. No skipped correlation is returned for pair 4.

We illustrate the use of the toolbox with the Anscombe’s (1973) quartet (Figures 1 and 2). For each pair of variables, standard Pearson’s and Spearman’s correlations were computed with their skipped-correlation counterparts, as well as the 20% percentage-bend correlation.

To compute skipped-correlations, first we estimate the robust center of the data cloud. Because a single outlier can result in the bivariate mean giving a poor reflection of the typical response, one relies here on the minimum covariance determinant (MCD) estimator, which is a robust estimator of multivariate location and scatter (Rousseeuw, 1984; Rousseeuw and Van Drissen, 1999; Hubert et al., 2008). The *skipped_correlation.m* function computes the MCD by calling the LIBRA toolbox (Verboten and Hubert, 2005 – free access at http://wis.kuleuven.be/stat/robust/LIBRA/LIBRA-home), which is distributed with the correlation toolbox under an academic public license. Second, outliers are identified using a projection technique: data points are orthogonally projected onto lines joining each data point to the robust estimate of location and outliers among projected data points are detected using the box-plot rule, which relies on the interquartile range (Frigge et al., 1989; Carling, 2000). Finally, Pearson’s and Spearman’s correlations and associated *t*-values are computed on the remaining data. The empirical *t*-values are compared to a critical *t*-value determined via simulations (Wilcox, 2012a,b). The usual critical value is technically unsound and should not be used because it does not take outlier removal into consideration; the critical values implemented in the toolbox ensure good control of the type I error rate.

To compute the percentage-bend correlation, a specified percentage of marginal observations deviating from the median are down weighted. Pearson’s correlation is then computed on the transformed data. A skipped correlation is a robust generalization of Pearson’s r by measuring the strength of the linear association, ignoring outliers detected by taking into account the overall structure of the data. In contrast, the percentage-bend correlation only protects against outliers associated with the marginal distributions. Under normality, the percentage-bend and Pearson’s correlations have very similar values, but these values can differ markedly as soon as there is deviation from normality (Wilcox, 1994).

The toolbox also computes percentile bootstrap 95% CIs for each correlation. For Pearson’s, Spearman’s, and percentage-bend correlations, pairs of observations are resampled with replacement and their correlation values obtained. For skipped-correlations, the data after outlier removal are resampled, before computing correlation values^1^. Correlation values are then sorted and the 2.5 and 97.5 percentiles obtained to yield a 95% CI. CIs provide an alternative way to test the null hypothesis. If the CI encompasses 0, then the null hypothesis of independence cannot be rejected. This is of particular interest when a correlation is declared significant (e.g., *p*-value < 0.05), because the *t*-test assumes independence between variables, which implies homoscedasticity. If there is heteroscedasticity, the *t*-test uses an incorrect estimate of the standard error. The significance of a correlation can therefore be largely affected by heteroscedasticity even though variables are not truly correlated. The toolbox thus also provides a rejection of the null hypothesis based the percentile bootstrap CI, because it is less sensitive to heteroscedasticity than the traditional *t*-test.

### Monte-Carlo simulations: False positives, effect sizes, and power

**Figure 3:**
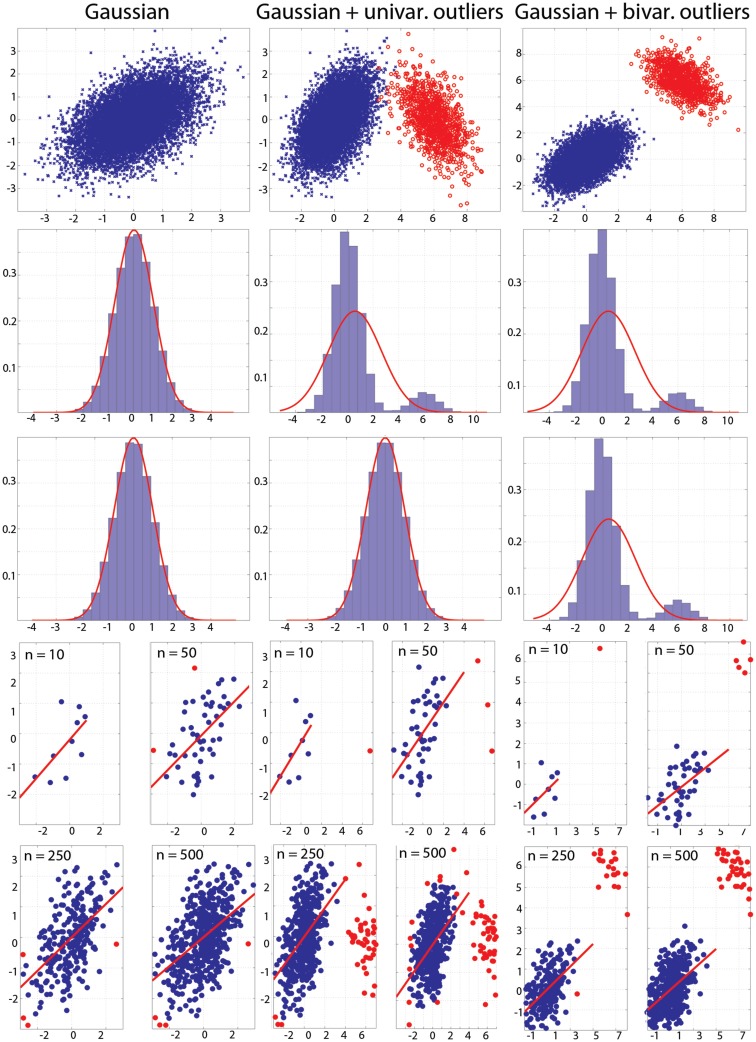
**Populations used in the simulations**. Top: populations with effect sizes of 0.5. Middle: marginal histograms for these populations. Bottom: examples of draws with sample sizes *n* = 10, 50, 250, and 500. Red dots mark bivariate outliers identified using the box-plot rule on project data.

To assess the sensitivity of the different correlation methods, we ran several simulations in which we recorded the actual correlation value (effect size) and the number of times the null hypothesis of independence was rejected (false positive rate and power). In the first simulation, a parent bivariate normal (*N* ∼ 0, 1) distribution of 10 million data points was generated (Figure 3, left column). For one Monte-Carlo run, 500 pairs of observations were randomly selected from the parent distribution. Using these 500 pairs, Pearson’s, Spearman’s, 20% bend and skipped-correlations were computed for sample sizes *n* = 10, 20, 30, 40, 50, 60, 80, 100, 150, 200, 250, 300, 400, and 500. The procedure was replicated 10,000 times (i.e., 10,000 independent samples of 500 pairs were taken from the parent population). The whole process was then repeated for parent populations in which the correlation values ranged from 0 to 1 with steps of 0.1. To generate Gaussian data with outliers, we generated one million data points from a parent bivariate normal distribution with a correlation value that was the negative of that in the first population. The center of this second population was positioned such that observations would be either marginal outliers for one variable (bivariate mean = [6, 0], Figure 3, middle column) or both (bivariate mean = [6, 6], Figure 3, right column – in this case thus also bivariate outliers). For each sample size, 10% of data were substituted by outliers taken at random from the outlier population: 1 outlier out of 10, 2 outliers out of 20, and so on.

To investigate effect sizes, we first tested if the correlations differed from the true population value. Differences between observed correlation values (*r*) and the true one (ρ) were computed, and Bonferroni adjusted percentile CIs were obtained (95% CI adjusted for the 14 sample sizes = 99.9964% CI). If 0 was not included in the 99.9964% CI, the method did not estimate the true correlation value. Second, we compared (i) Pearson’s correlation against Spearman’s, a 20% bend, and skipped Pearson’s correlations, and (ii) Spearman’s correlation against skipped Spearman’s correlation. A percentile bootstrap on the median differences was computed and adjusted for the 14 sample sizes (α set to 0.05/14 = 0.36%): the results from two correlations differed significantly if the CI of median differences did not contain zero.

To evaluate the false positive rate and power, the average number of times the null hypothesis was rejected was computed. The different correlation techniques were then compared for each sample size based on their binomial distributions (accept/reject H0) using a method for discrete cases with adjustment for multiple comparisons (Kulinskaya et al., 2010).

## Results

### Illustration with the Anscombe’s quartet

As put forward by Anscombe (1973), plotting the data is an important part of any statistical analysis (Figure 1, left column). For the reader not aware of this data set, it is important to know that for each of the four pairs of variables *X* and *Y*, the mean of *X* is 9, the variance of *X* is 11, the mean of *Y* is 7.5, the variance of *Y* is 4.12, and Pearson’s correlation between *X* and *Y* is always 0.81. Despite these identical first order statistical properties and identical correlation values, the nature of the relationships between *X* and *Y* differs widely. For pair 1, inspection of the scatterplot and distributions suggests close to normally distributed data with no obvious outlier. Pair 2 shows a non-linear and non-monotonic relationship and data are not normally distributed. Pair 3 shows a strict linear relationship and 1 marginal outlier. Finally, pair 4 shows no relationship and 1 bivariate outlier.

The Henze–Zirkler test for multivariate normality confirmed visual inspection: only pair 1 is normally distributed (HZ = 0.1, *p* = 0.99), whilst the other pairs deviate from the bivariate normal distribution (pair 2 HZ = 0.6, *p* = 0.036; pair 3 HZ = 1.04, *p* = 0.002; pair 4 HZ = 1.06, *p* = 0.002). The outlier detection function implemented in the toolbox relies on three methods: the box-plot rule, as used in the skipped correlation function, the median absolute deviation (MAD)-median rule (Hall and Welsh, 1985), and the S-estimator deviation (Rousseeuw and Croux, 1993). Results from the box-plot rule show no univariate or bivariate outliers in pairs 1 and 2, one univariate outlier pair 3, and one univariate and simultaneously bivariate outlier in pair 4 (Figure 1, middle columns). For pair 1, other methods gave the same result. For pair 2, both the MAD-median rule and S-outlier methods identified the first two points as univariate outliers in *Y*. In addition, the MAD-median rule identified the first and last points as bivariate outliers, whereas the S-outlier method identified the first and the last two points as bivariate outliers. This illustrates the difficulty of spotting bivariate outliers because of the trade off between specificity (true negatives) and sensitivity (true positives – Appendix). For pairs 3 and 4, the MAD-median rule and the S-outlier method also flagged the extreme points as outliers. Finally, tests of variance homogeneity revealed that variances differed significantly in pairs 1 and 2, but not in pairs 3 and 4 (Figure 1, right column). Heteroscedasticity, rather than true association, could thus have caused significant correlations for pairs 1 or 2 (Wilcox, 1991; Wilcox and Muska, 2001). In comparison, Levene’s tests failed to reject the null hypothesis of homoscedasticity for all pairs [pair 1 *F*(1,20) = 3.5, *p* = 0.07; pair 2 *F*(1,20) = 3.39, *p* = 0.08; pair 3 *F*(1,20) = 4.15, *p* = 0.055; pair 4 *F*(1,20) = 0.17, *p* = 0.68]. This is explained by Levene’s test lack of power: the test is based on the distance between each point from the mean, which by definition is affected by outliers.

As designed by Anscombe, Pearson’s correlation is fooled by outliers and, for each pair, a significant correlation of *r* = 0.81 is observed (Table 1; Figure 2). Importantly, bootstrap CIs are also sensitive to outliers and suggest significant correlations too. Spearman’s correlations performed slightly better, showing no association in pair 4. In addition, the bootstrap CI in pair 2 shows no evidence for a significant correlation, suggesting that the observations are not linearly related but show dependence. The 20% percentage-bend correlation gives better results than Pearson’s or Spearman’s correlations. For normal data (pair 1), it performs similarly to Pearson’s correlation. For a non-linear relationship (pair 2), like Spearman, the 20% percentage-bend correlation returns significant results but the bootstrap CI does not. With a univariate outlier (pair 3), it returns the exact correlation. Finally, it shows no significant results for pair 4. Here the bootstrapped CI suggests a significant result, which is explained by the use of valid resamples (i.e., resamples cannot be composed of a unique value) to compute CIs in our algorithm, that is, for each bootstrap, the single outlier in *Y* was always present. Inspection of the data plot nevertheless reveals that the bootstrap did not perform well. This again illustrates that the bootstrap, on it’s own, does not protect against outliers, although it can attenuate their effect. The skipped correlation returned the same results as Pearson’s and Spearman’s correlations because the box-plot rule did not detect the bivariate outliers in pairs 1, 2, and 3. The skipped correlation failed to provide any output for pair 4, because, once the outlier is removed, the remaining points are aligned with the same *X* value and it is thus impossible to compute any correlation. We would indeed expect robust analyses not to find any association for such data.

**Table 1:** **Correlation results with their 95% CIs for the Anscombe’s quartet**.

	Pair 1	Pair 2	Pair 3	Pair 4
Pearson	*r* = 0.81, *p* = 0.002	*r* = 0.81, *p* = 0.002	*r* = 0.81, *p* = 0.002	*r* = 0.81, *p* = 0.002
	*h* = 1, CI [0.59, 0.95]	*h* = 1, CI [0.48, 0.96]	*h* = 1, CI [0.71, 1]	*h* = 1, CI [0.75, 0.95]
Spearman	*r* = 0.81, *p* = 0.002	*r* = 0.69, *p* = 0.01	*r* = 0.99, *p* ∼ 0	*r* = 0.5, *p* = 0.1
	*h* = 1, CI [0.49, 0.97]	*h* = 0, CI [−0.009, 1]	*h* = 1, CI [0.99, 1]	*h* = 1, CI [0.5, 0.8]
20% bend	*r* = 0.81, *p* = 0.002	*r* = 0.8, *p* = 0.0029	*r* = 1, *p* = 0	*r* = 0.22, *p* = 0.49
	*h* = 1, CI [0.43, 0.96]	*h* = 0, CI [−0.06, 0.99]	*h* = 1, CI [0.89, 1]	*h* = 1, CI [0.2, 0.79]
Skipped Pearson	*r* = 0.81, *h* = 1	*r* = 0.81, *h* = 1	*r* = 0.81, *h* = 1	*r* = NaN, *h* = 0
	*h* = 1, CI [0.54, 0.95]	*h* = 1, CI [0.51, 0.96]	*h* = 1, CI [0.74, 1]	*h* = 0, CI [NaN NaN]
Skipped Spearman	*r* = 0.81, *h* = 1	*r* = 0.69, *h* = 0	*r* = 0.99, *h* = 1	*r* = NaN, *h* = 0
	*h* = 1, CI [0.4, 0.97]	*h* = 1, CI [0, 1]	*h* = 1, CI [0.9, 1]	*h* = 0, CI [NaN NaN]

### Monte-Carlo simulations

Figure 3 illustrates the populations used in the simulations. The population in the top left subplot had a Pearson’s correlation of 0.5. It is important to see that outliers in the bivariate space (illustrated in red) can be observed even though univariate distributions are perfectly normal (case 2). Outliers can be important for the process under study, but given the goal of characterizing the bulk of the data, they can result in misleading conclusions. As illustrated at the bottom of Figure 3, outliers can be present even in data from a normal population, because the sample itself might not be normal.

#### Zero-correlation and false positive error rate

##### Gaussian data

**Figure 4:**
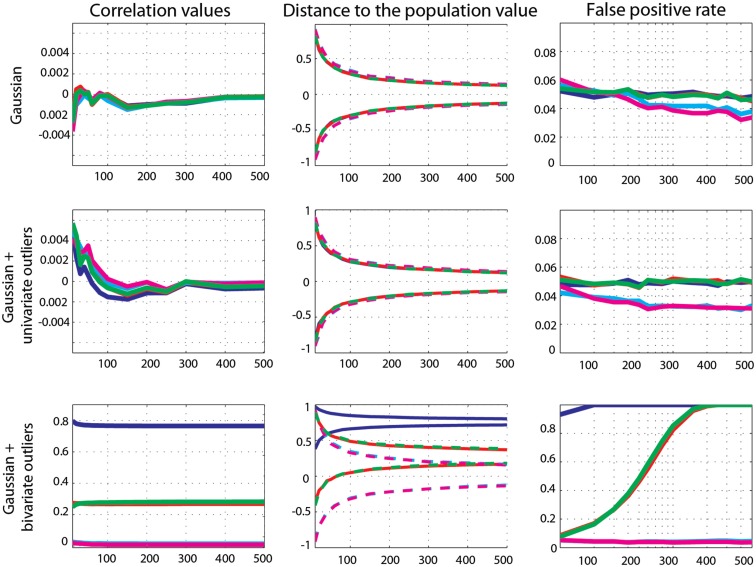
**Effect sizes and false positive error rates for Gaussian data with zero-correlation**. From left to right are displayed: the mean correlation values; the 99.99% CIs (i.e., corrected for the 14 sample sizes) of the distance to the zero-correlation in the simulated Gaussian population; the false positive rate for Pearsons’ (blue), skipped Pearson’s (cyan), Spearman’s (red), skipped Spearman’s (magenta), and 20% bend (green) correlations for each type of simulation (Gaussian only, with univariate outliers, and with bivariate outliers). The *Y*-axis scales are different for data with bivariate outliers.

Zero-correlation was well estimated by all methods: all correlation values were close to 0 and the 99.99% CIs of all methods included 0 (Figure 4). Comparisons of methods showed no significant differences between Pearson’s and Spearman’s (0.1 < *p* < 0.8) and the 20% percentage-bend (0.24 < *p* < 0.99) correlations. Pearson’s correlations and skipped Pearson’s correlations showed small (∼0.001) but significant differences for *n* = 10–100 (*p* = 0) and did not differ for *n* > 100 (0.01 < *p* < 0.59). Similarly, the standard and skipped Spearman’s correlations differed significantly for *n* = 10–100 (*p* = 0) and did not differ for *n* > 100 (0.05 < *p* < 0.69). The false positive rate was well controlled by all methods, with values close to the 5% nominal level. Across sample sizes, the average false positive error rate for Pearson’s correlation was 4.9% (min 4.7, max 5.2), for Spearman’s correlation 4.9% (min 4.5, max 5.6), for the 20% percentage-bend correlation 4.9% (min 4.6, max 5.4), for the skipped Pearson’s correlation 4.4% (min 3.6, max 5.6), and for skipped Spearman’s correlation 4.1% (min 3.2, max 6). Comparison of the binomial distributions (significant/non-significant results) found that Pearson’s correlations did not differ significantly from Spearman’s (0.19 < *p* < 0.98) and from the 20% percentage-bend (0.23 < *p* < 0.88) correlations. From *n* = 10–100, the false positive rate was not different between standard- and skipped-correlations (Pearson 0.004 < *p* < 0.8; Spearman 0.001 < *p* < 0.9). However, the false positive rates did differ for larger sample sizes (Pearson 0.001 < *p* < 0.009; Spearman 0.001 < *p* < 0.002) such that skipped-correlations were more conservative.

##### Marginal outlier data

Again, zero-correlation was well estimated by all methods as all correlation values were close to 0. The 99.99% CIs of all methods included 0 (Figure 4). Comparison of methods showed no significant differences between Pearson’s and Spearman’s (0.15 < *p* < 0.95), the 20% percentage-bend (0.08 < *p* < 0.95), or the skipped Pearson’s (0.03 < *p* < 0.99) correlations. No significant differences were observed between Spearman’s and skipped Spearman’s correlations (0.05 < *p* < 0.84). The false positive rate was well controlled by Pearson’s correlation (average false positive error rate 4.8%, min 4.7, max 5), Spearman’s correlation (4.9%, min 4.5, max 5.3), and the 20% percentage-bend correlation (4.9%, min 4.6, max 5.1). Skipped-correlations were slightly conservative with an average false positive error rate, for the skipped Pearson’s correlation, of 3.4% (min 3, max 4) and, for skipped Spearman’s correlation, of 3.3% (min 3, max 4). Comparison of the binomial distributions revealed that Pearson’s correlation did not differ from Spearman’s (0.07 < *p* < 0.98) and the 20% percentage-bend (0.27 < *p* < 0.99) correlations. However, for *n* > 20, the false positive rates were significantly smaller for skipped-correlations (Pearson 0.001 < *p* < 0.004; Spearman 0.001 < *p* < 0.002).

##### Bivariate outlier data

Only skipped correlation methods estimated well zero-correlation. On average, Pearson’s correlation was *r* = 0.77 (min 0.76, max 0.079) and the 99.99 CIs never included 0. Spearman and the 20% percentage-bend correlations showed similar results with averaged correlations of 0.269 (min 0.268, max 0.273) and 0.275 (min 0.25, max 0.282), and CIs included 0 for *n* = 10–60 only. In contrast, skipped Pearson’s and Spearman’s correlations were close to 0 with average correlations of 0.017 (min 0.014, max 0.026) and 0.011 (min 0.008, max 0.02). Their CIs always included 0. Pearson’s correlation estimates were significantly larger than estimates from Spearman’s (*p* = 0), 20% percentage-bend (*p* = 0), and skipped Pearson’s correlations (*p* = 0). Similarly, Spearman’s correlations were significantly larger than their skipped counterparts (*p* = 0). The false positive error rate was close to or equal to 100% for Pearson’s correlations. For Spearman’s and the 20% percentage-bend correlation, it increased from 8.53% for *n* = 10 to 100% for *n* = 250. In contrast, the false positive error rate of skipped-correlations stayed close to the nominal level of 5% (average rate for skipped Pearson’s correlations 4.3%, min 3.7, max 5.1; average rate for skipped Spearman’s correlations 4%, min 3.5, max 5.2). Comparison of the binomial distributions revealed that Pearson’s correlations differed significantly from all of the other methods (*p* = 0.001), except Spearman’s and the 20% percentage-bend correlations for *n* > 300, where they also provided 100% of false positives.

#### Effect sizes and power

##### Gaussian data

**Figure 5:**
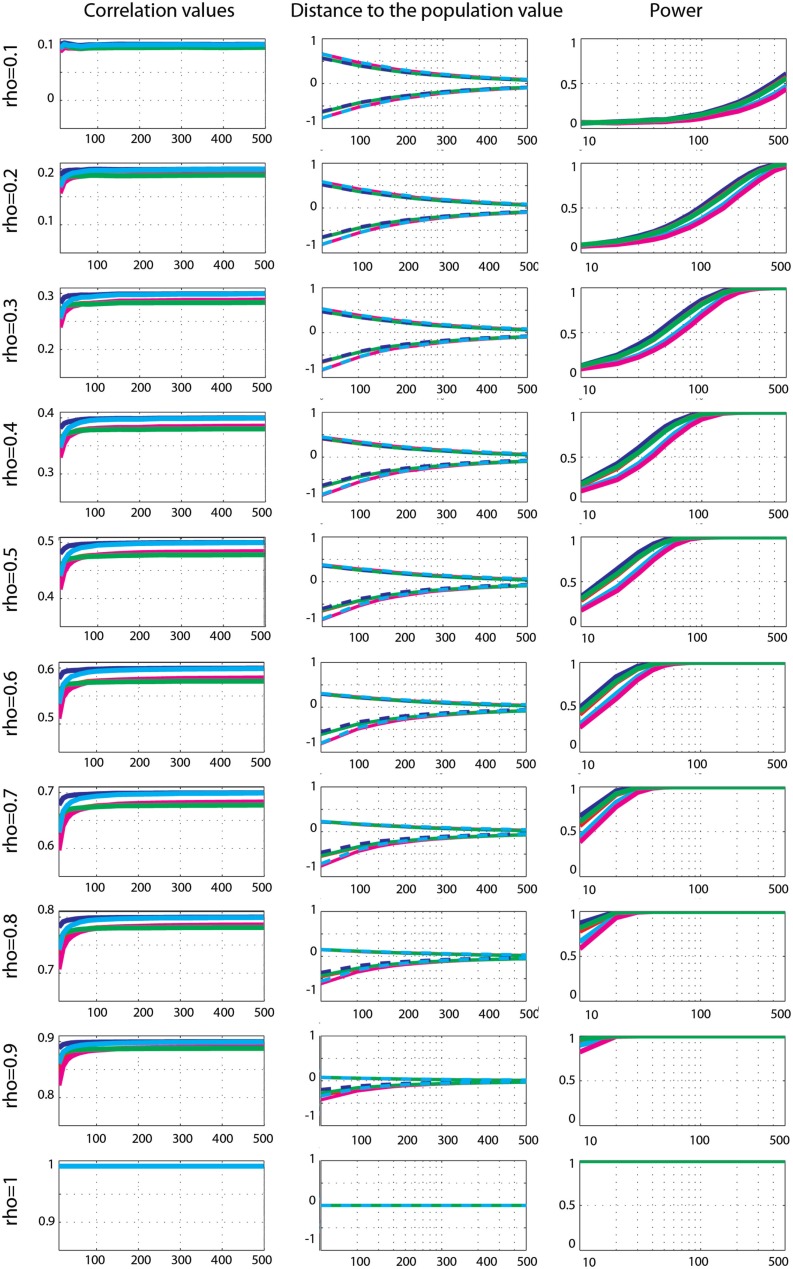
**Effect sizes and power for Gaussian data**. From left to right are displayed: the mean correlation values; the 99.99% CIs (i.e., corrected for the 14 sample sizes) of the distance to the correlation in the simulated Gaussian population; the power for Pearson’s (blue), skipped Pearson’s (cyan), Spearman’s (red), skipped Spearman’s (magenta), and 20% bend (green) correlations for each effect size (from top *r* = 0.1 to bottom *r* = 1).

Effect sizes for Gaussian data (Figure 5) were well captured by all methods: the 99.99% CIs of the difference to the true correlations all encompassed 0. Comparisons of methods nevertheless revealed differences, with Pearson’s correlation being the best method of all. Compared to Spearman’s correlation, Pearson correlation was significantly higher (closer to the true value) for 0.1 < ρ < 0.9 (*p* = 0), with differences from +0.004 to +0.03. The same pattern was observed when compared to the 20% percentage-bend correlation (*p* = 0), except for ρ = 0.1 and *n* = 10 (*p* = 0.18), with differences from +0.001 to +0.02. When compared to skipped Pearson’s correlation, significant differences were observed from ρ > 6, *n* > 400 to ρ = 0.9, *n* > 100 (0 < *p* < 0.002 – differences ranging from +0.006 to +0.001). For smaller correlation values and sample sizes there were no significant differences (0.1 < *p* < 1). A similar pattern of results was observed when comparing Spearman’s correlation to skipped Spearman’s correlation. Significant differences were observed from ρ > 3, *n* > 400 to ρ = 0.9, *n* = 80 (0 < *p* < 0.002 – from −0.001 to +0.002). For smaller correlation values and sample sizes there were no significant differences (0.1 < *p* < 1). For all comparisons, there were no significant differences when ρ (the true correlation value) was equal to 1.

Power analyses showed similar trends for all techniques, with maximum power for Pearson’s correlations and minimum power for skipped Spearman’s correlations. In general, power increased up to 100% as a function of the sample size except for *r* = 0.1. Comparison between methods revealed significantly stronger power for Pearson’s correlation compared to Spearman’s correlation (max +10%, 0.001 < *p* < 0.003), from high correlations and small sample sizes (ρ > 0.3, *n* < 150), to low correlations and large sample sizes (ρ < 0.2, *n* > 250). For small correlation values and small sample sizes or large correlation values and large sample sizes, the two methods had similar power (0.004 < *p* < 0.99). The same results (with the exception of 6 comparisons out of 126) were observed when comparing Pearson’s correlations to the 20% percentage-bend correlation (max difference +6.4%). Power comparison between Pearson’s correlation and skipped Pearson’s correlation showed significant differences (max difference +23% 0.003 < *p* < 0.003) for all effect sizes as a function of the sample size. Pearson’s correlation was more powerful than skipped Pearson’s correlation at increasing sample sizes as r decreased (ρ < 1, *n* = 30; ρ < 0.9, *n* = 40; ρ < 0.8, *n* = 50; ρ < 0.7, *n* = 60; ρ < 0.6, *n* = 60; ρ < 0.5, *n* = 100; ρ < 0.4, *n* = 150; ρ < 0.3, *n* = 250; ρ < 0.2, *n* = 300); however, for large effect sizes and large sample sizes, the two techniques did not differ (0.01 < *p* < 0.99). The same results (with the exception of 4 comparisons out of 126) were observed when comparing Spearman’s correlations to the skipped Spearman correlation (max difference +19%).

##### Marginal outlier data

**Figure 6:**
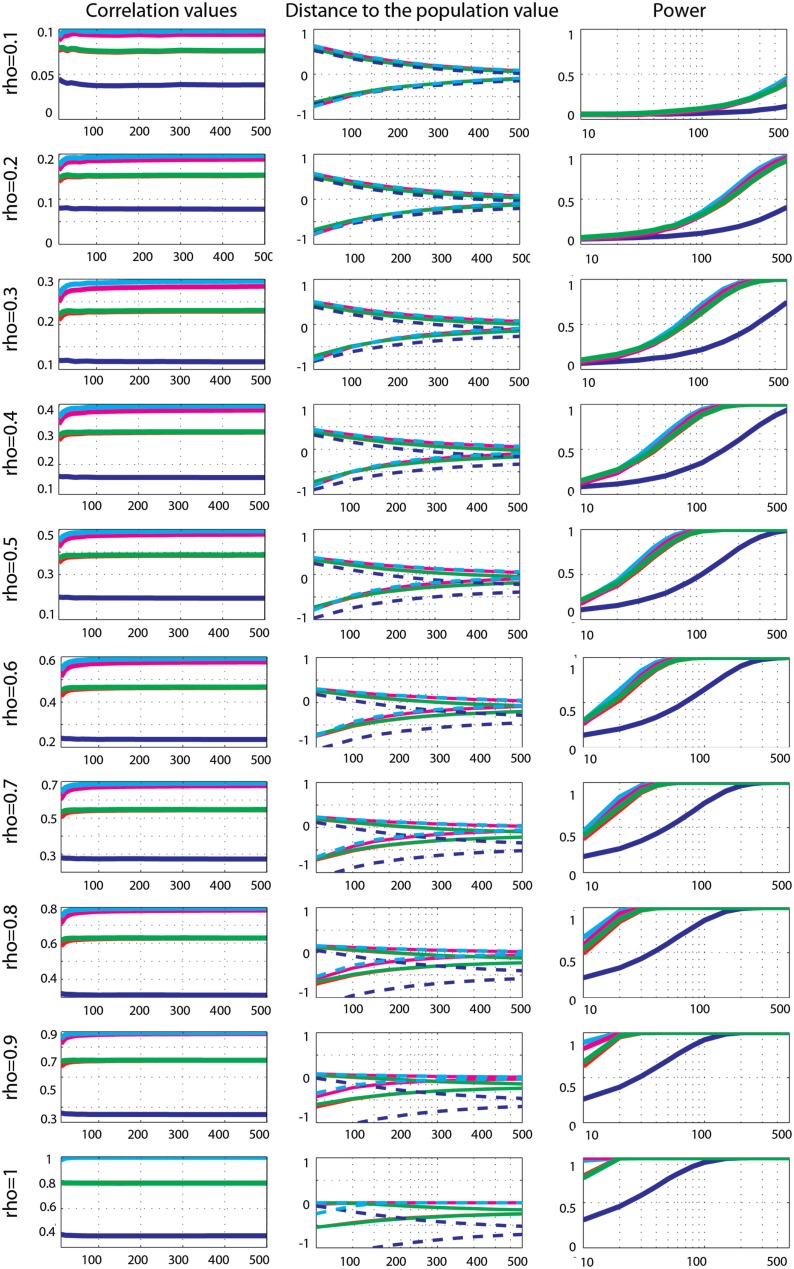
**Effect sizes and power for Gaussian data contaminated by 10% of marginal outliers**. From left to right are displayed: the mean correlation values; the 99.99% CIs (i.e., corrected for the 14 sample sizes) of the distance to the correlation in the simulated Gaussian population contaminated by univariate outliers; the power for Pearson’s (blue), skipped Pearson’s (cyan), Spearman’s (red), skipped Spearman’s (magenta), and 20% bend (green) correlations for each effect size (from top *r* = 0.1 to bottom *r* = 1). In column one, the scales of the mean correlation values differ.

Effect sizes for Gaussian data contaminated by 10% of marginal outliers (Figure 6) showed that Pearson’s and Spearman’s correlations estimated poorly the true correlations, whereas skipped estimators always estimated properly ρ (all 99.99% CIs included 0). Pearson’s correlations underestimated ρ most of time – the 99.99% CIs included 0 for only 30% of cases: for ρ = 0.1; ρ = 0.2, *n* < 250; ρ = 0.3, *n* < 100; ρ = 0.6 *n* < 60; ρ = 0.5, *n* < 40; ρ = 0.6, *n* < 30; ρ = 0.7 and 0.8, *n* < 20. This shows that the more the outliers deviated from the population, the less Pearson’s correlation could estimate the true effect size. A similar pattern of results was observed for Spearman’s correlations although estimates were better, with 59% of correct cases. The 99.99% CIs included 0 for ρ = 0.1, 0.2, and 0.3; ρ = 0.4, *n* < 400; ρ = 0.5, *n* < 200; ρ = 0.6, *n* < 150; ρ = 0.7, *n* < 80; ρ = 0.8, *n* < 40; ρ = 0.9, *n* < 30; ρ = 1, *n* < 20. The 20% percentage-bend correlation matched closely Spearman’s estimates with 99.99% CIs including 0 in 60% of cases. However, remember that although the percentage-bend correlation is restricted to linear relationships, it does not estimate ρ. Comparisons of methods revealed that Pearson’s correlations were always lower than Spearman’s (*p* = 0), 20% percentage-bend (*p* = 0), and skipped Pearson’s (*p* = 0) correlations. Similarly Spearman’s correlations were always significantly different from skipped Spearman’s correlations (*p* = 0).

Power curves revealed that when effect sizes were well estimated, Spearman’s (0.001 < *p* < 0.002), 20% percentage-bend (*p* = 0.001), and skipped Pearson’s (*p* = 0.001) correlations were more powerful than the standard Pearson’s correlation. Similarly, the skipped Spearman’s correlation was more powerful than the standard Spearman correlation in most cases (0.001 < *p* = 0.003).

##### Bivariate outlier data

**Figure 7:**
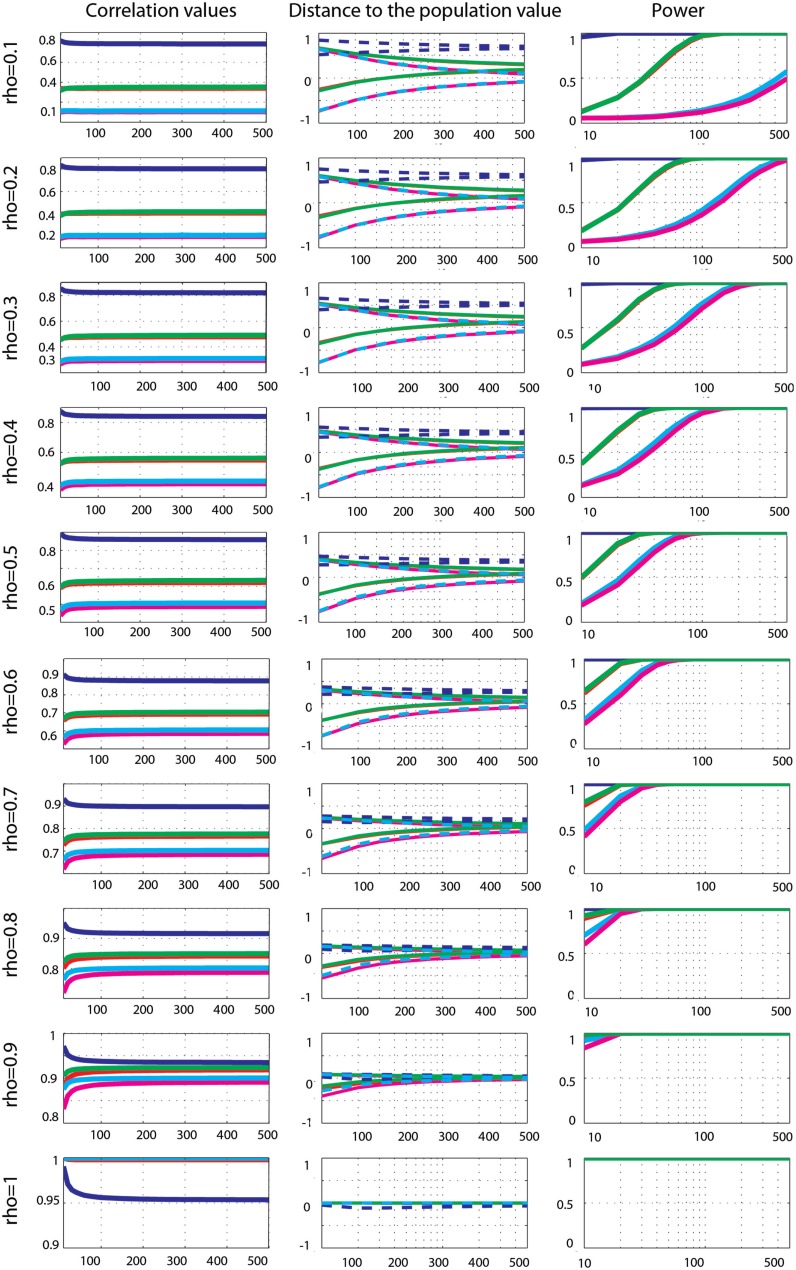
**Effect sizes and power for Gaussian data contaminated by 10% of bivariate outliers**. From left to right are displayed: the mean correlation values; the 99.99% CIs (i.e., corrected for the 14 sample sizes) of the distance to the correlation in the simulated Gaussian population contaminated by bivariate outliers; the power for Pearson’s (blue), skipped Pearson’s (cyan), Spearman’s (red), skipped Spearman’s (magenta), and 20% bend (green) correlations for each effect size (from top *r* = 0.1 to bottom *r* = 1). In column one, the scales of the mean correlation values differ.

Effect sizes for Gaussian data contaminated by 10% of bivariate outliers (Figure 7) showed that Pearson’s and Spearman’s correlations estimated correlations poorly, whereas skipped-correlations performed well. Pearson’s correlation never estimated well ρ except for ρ = 0.9, 10 < *n* < 150 (in total only 5% of cases). Spearman’s correlation was less sensitive to bivariate outliers (in total 49% of cases were correct) and correct estimates were observed from small correlation values and small sample sizes to high correlation values and larger sample sizes (ρ = 0.1, *n* < 40; ρ = 0.2, *n* < 50; ρ = 0.3 and 0.4, *n* < 80; ρ = 0.5 and 0.6, *n* < 150; ρ = 0.7, *n* < 200; ρ = 0.8, *n* < 300; ρ = 0.9, *n* < 500; ρ = 1 *n* < 20). The 20% percentage-bend correlation did not estimate well the population correlation either (in total 52% of cases encompassed ρ), with again results very similar to those of Spearman’s correlation. In contrast to standard methods, skipped Pearson’s and Spearman’s correlations always properly estimated ρ. Comparisons of methods revealed that Pearson’s correlations were always significantly different from Spearman’s (*p* = 0), 20% percentage-bend (*p* = 0), and skipped Pearson’s (*p* = 0) correlations (higher for 0.1 < ρ < 0.9 and lower for ρ = 1). Similarly, Spearman’s correlation always differed significantly from skipped Spearman’s correlation (*p* = 0).

The power of Pearson’s correlation did not differ from that of other methods for the few correct estimations (*p* > 0.003). More interestingly, comparisons of Spearman’s versus skipped Spearman’s correlations show that for low ρ, the standard Spearman approach was more powerful when estimates were correct (*p* = 0.003).

## Discussion

When data were normally distributed, Pearson’s correlation was the best method, estimating best the true effect sizes and showing more power. Robust alternatives still estimated properly the true effect sizes with slight differences (from −0.001 to −0.02 for the 20% percentage-bend correlation and from −0.006 to −0.001 for the skipped Pearson’s correlation). Those results can be explained by the fact that those robust techniques down-weight or remove data points from the samples being drawn. As a consequence, they also have less power (at most −6% for the 20% percentage-bend correlation and −23% for the skipped Pearson’s correlation). However, the assumption of normality rarely holds (e.g., Micceri, 1989) and when it is not met, using Pearson’s or Spearman’s correlations can lead to serious errors. In our simulations, both techniques grossly overestimated or underestimated the true effect sizes depending on the position of outliers relative to the population, whereas their skipped counterparts performed well in all the cases analyzed.

**Figure 8:**
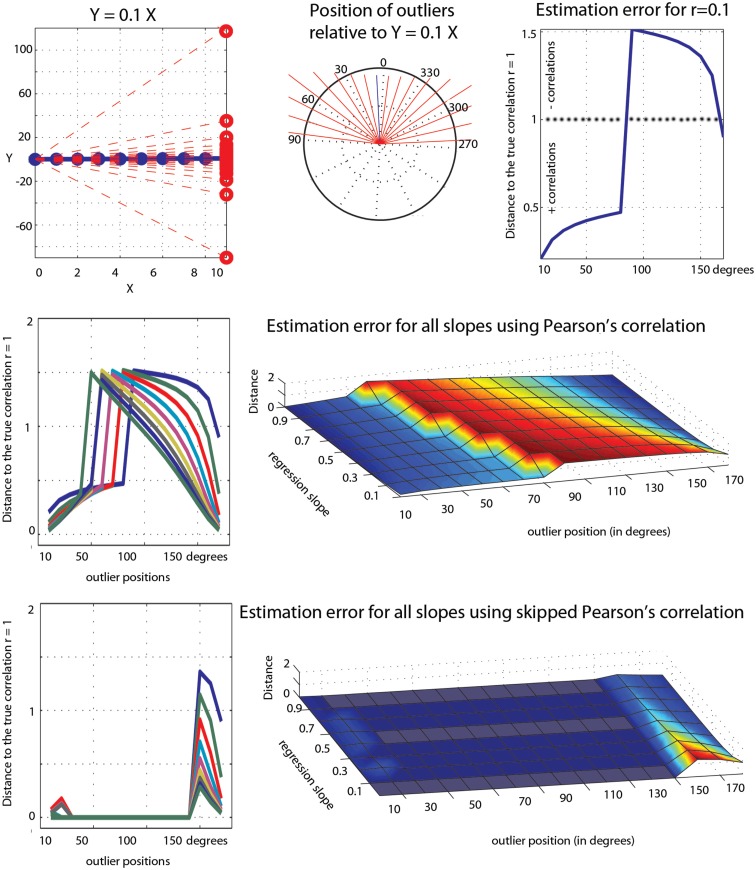
**Illustration of the effect of a single outlier among 10 data points on Pearson’s correlation**. At the top is illustrated the outlier values (red circles in the left plot), their positions in the bivariate space (the end of each red line in the polar plot) relative to the regression line *Y* = 0.1*X*, and the error in Pearson’s estimates (1 – observed correlation). The middle row shows similar results for all slopes (from 0.1 to 0.9). The bottom row shows the results from the skipped correlation.

The first point to consider is the estimation of the true effect sizes in the context of marginal and bivariate outliers. In our simulations, Pearson’s and Spearman’s correlations failed most of the time but occasionally estimated properly ρ. These accurate estimations should not be taken as an indication of the robustness of the methods, but simply an illustration of the effect of the position of outliers. In the case of univariate outliers, outliers were located in such a way that there positions were between −0.3 and −9.94° relative to the population of interest. As a consequence, Pearson’s and Spearman’s correlations always underestimated ρ, being attracted toward [6, 0], the center of the outlier population. In the case of bivariate outliers, outliers were located in such a way that there positions were between +0.4 and +13.4° relative to the population of interest. As a consequence, Pearson’s and Spearman’s almost always overestimated ρ (the exception being ρ = 0.9 where the 2 population were aligned), being attracted toward [6, 6]. To further illustrate this effect of the position of outliers, consider the toy example in Figure 8. The data are similar in spirit to pair 3 from Anscombe’s quartet. We first created 10 points perfectly aligned (Pearson’s *r* = 1) and then rotated the regression line by steps of 10° and substituted the last point of the initial data by the last point of the rotated data. Results show that, as the single outlier gets farther and father away from the initial value (i.e., father away in the bivariate space), Pearson’s estimates become overly sensitive to it. Estimation errors varied up to 1.5 unit, i.e., a single outlier could reduce the correlation by 50% or completely reverse it (equivalent to −150%). An extreme case in this toy example is for *Y* = 0.2*X* and a 80° rotation of the last point; this data point goes from [9, 1.8] to [9, −393] and r changes from *r* = 1 to −0.51. Of course, anybody looking at the data would spot this point as an outlier. Skipped-correlations detect and remove such data point whilst accounting for the deletion when testing for significance. Removing data points and running the analysis without accounting for the removal is not good practice because the standard error estimates would be incorrect and can substantially alter a test statistic. Ignoring this issue when dealing with correlations can result in poor control over the probability of a Type I error (Wilcox, 2012a). In our toy example, the outlier detection fails for small deviations of the outlier in the bivariate space (10, 20, 150, 160, and 170° – Figure 8) but identify correctly the outlier in all other cases such that the final correlation is 1.

The second point to consider is the power of each method. It has been argued that skipped correlation can lack power compared to Pearson’s correlation (Schwarzkopf et al., 2012). Our simulations show that this is the case only if the data are perfectly normal. In contrast, when data contain outliers, skipped Pearson’s correlation can be more powerful. In our simulations, the only cases in which Pearson’s correlation clearly outperformed the skipped Pearson’s correlations was when the effect sizes were largely overestimated due to outliers (see e.g., Figure 7), which of course make its use inappropriate^2^. Because in many cases data do not conform to normality (e.g., Micceri, 1989), Pearson’s skipped-correlations seem to provide a good alternative to classic techniques, particularly in the presence of bivariate outliers. In case of departure from normality, Spearman’s correlation is often advocated as an alternative to Pearson’s correlation. Our simulations showed that when outliers contaminate data, Spearman’s correlation indeed performs better than Pearson’s correlation and can have stronger power. Estimated correlations can however also be strongly biased. An alternative to Spearman’s correlation seems to be the (20%) percentage-bend correlation. In our simulations it closely matched Spearman’s and skipped Spearman’s correlations power in the presence of marginal outliers. Thus, although its coefficient cannot be interpreted as a reflection of ρ, it provides an alternative to test the significance of linear correlations, especially if outliers are not detected in the bivariate space.

The last point to consider is the type I error rate. Schwarzkopf et al. (2012) also suggested that skipped-correlations have an inflated false positive rate. Results from our simulations show otherwise: skipped-correlations are in fact slightly conservative with normal data or data contaminated by marginal outliers and achieve a type I error rate at the 5% nominal level when bivariate outliers contaminate the data, which agrees with previous observations (Wilcox, 2004). It is however possible that the type I error rate increases when outliers are in the margins of the population of interest, thus leading to large variance, as in the simulation by Schwarzkopf et al. (2012). We reproduced their simulation for *n* = 10 and computed Pearson’s correlation, Pearson’s correlation after removing outliers flagged by the MCD algorithms, and skipped Pearson’s correlation using the box-plot rule (the method used in the toolbox) or the MAD-median rule on projected data. For two independent normal variables *N* ∼ (0, 1) and one univariate outlier from *N* ∼ (0, 3), Pearson’s correlation showed a type I error rate of 0.048. If outliers were removed using the MCD algorithm, the type I error rate rose to 0.39. The standard skipped correlation, however, had a type I error rate of 0.051, whereas using the MAD-median rule led to a type I error rate of 0.14. When the outlier was taken from a bivariate distribution with covariance ([3, 4.5; 4.5, 9]), Pearson’s correlation showed a type I error rate of 0.15. If outliers were removed using the MCD algorithm, the type I error rate rose to 0.4. Again, the standard skipped correlation had a type I error rate of 0.054, whereas using the MAD-median rule led to a type I error rate of 0.16. Although our results are slightly different from Schwarzkopf et al. (2012), they suggest that the authors identified outliers using the MAD-median rule on projected data, which indeed leads to a high false positive rate. If the adjusted box-plot rule is used as in our toolbox and simulations, the nominal level is achieved. These last results demonstrate how critical it is to (i) properly identify and remove outliers, a job well performed by the projection method compared to the output from the MCD algorithm, (ii) use a method with high specificity (removing only outliers), like the adjusted box-plot rule compared to the MAD-median rule, and (iii) adjust the test of significance to take into account the dependencies among data points after removing outliers.

To conclude, we demonstrated that robust alternatives to standard correlation methods provide accurate estimates of the true correlations for normal and contaminated data with no or minimal loss of power and adequate control over the false positive rate. Given the range of possible data configurations, all scenarios cannot be tested but some recommendations can be drawn from our results. *First*, before computing any relationship, plot the data and run several outlier detection methods. If inspection of the scatter plot suggests a non-linear relationship (e.g., pair 2 of Anscombe’s quartet) or the marginal distributions suggest that the data are not normally (or close to normally) distributed, one should choose alternative methods to the ones considered in the present article. Indeed, the skipped Pearson correlation and the percentage-bend correlation are appropriate for linear relationships only, whereas the skipped Spearman correlation is also appropriate for monotonic relationships. Alternatively, for non-linear relationships, a generalization of Pearson’s correlation, called explanatory power, coupled with smoothers (non-parametric regression methods) provides a flexible approach to dealing with curvature (Wilcox, 2012a). Similarly, for non-Gaussian data, or non-linear relationships, or both, copulas offer a generalized approach to test for dependence (Sklar, 1959; Frees and Valdez, 1999): almost any correlated joint distribution can be modeled via marginal distributions and their copula, i.e., their link function. Copulas establish the dependence between variables and estimate the location of this dependency. In contrast, correlations estimate only average dependencies across the whole data range. *Second*, choose among methods given the data at hand and not given their results. For instance, use a percentage-bend correlation when univariate outliers are identified (e.g., Ancombe’s pair 3), or use a skipped-correlation when bivariate outliers are identified (e.g., Ancombe’s pair 4). *Third*, if a correlation method returns a significant result, check the variance homogeneity assumption using a bootstrap CI. This helps confirm that a significant result is due to a linear (or monotonic for Spearman’s correlation) association rather than heteroscedasticity. Also, the bootstrap is particularly useful when used in conjunction with robust estimators because resampling data with outliers necessarily leads to CIs either too large or too narrow. In the Anscombe’s quartet, bootstrapping the data still leads to significant results for pair 2 (a non-linear association) or pair 4 (no association) when used with Pearson’s correlation. *Finally*, always interpret correlation results by taking into account their effect sizes and bootstrap CIs.

## Conflict of Interest Statement

The authors declare that the research was conducted in the absence of any commercial or financial relationships that could be construed as a potential conflict of interest.

## Figures and Tables

**Figure A1:**
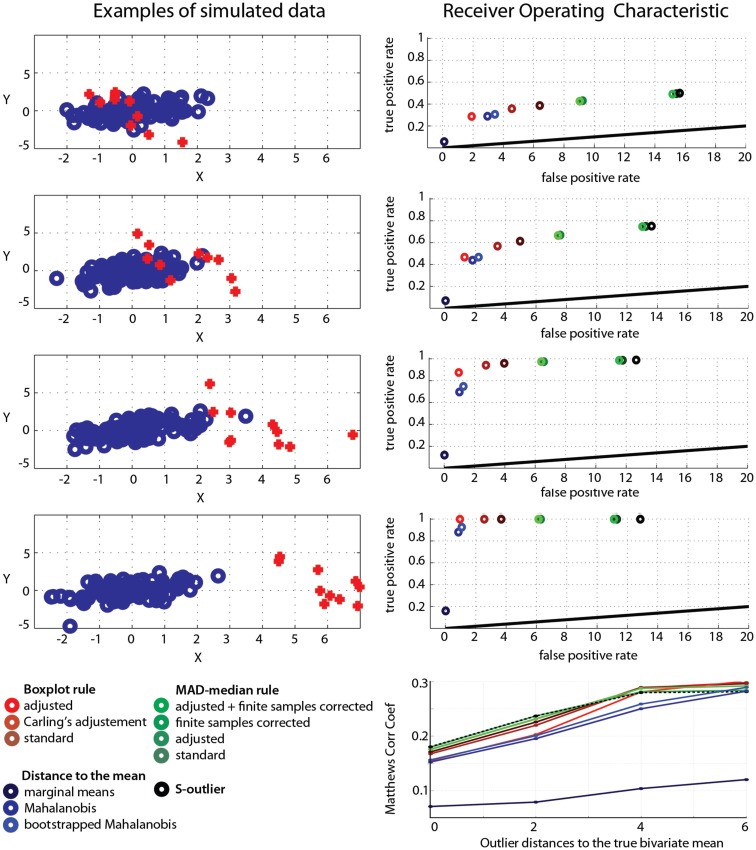
**Operating characteristics of outlier detection methods**. The left column illustrates the data generation process: each row shows different types of outliers, identified in red, and created by changing the amount of shift along the *X* direction. The right column shows the false positive rate (1-specificity) as a function of the true positive rate (sensitivity) for each method. Bottom right show the Matthews correlation coefficients. Red to brown: box-plot results; light green to dark green: MAD-median rule results; black: the S-outlier results; light to dark blue: deviations from the mean(s) results.

## Footnotes

^1^There are possible issues about the dependence among order statistics by removing outliers and bootstrapping rather than bootstrapping and re-run the whole skipped correlation. However, at the moment it is unclear which method is best.

^2^In Schwarzkopf et al. (2012) power is reported without formal tests of differences among methods and without considering effect sizes. It is thus unclear which conditions differed in their simulations.

## Appendix

There are various methods to detect outliers. In the context of skipped-correlations, one relies on the detection of univariate outliers among projected data points (points orthogonally projected onto lines joining each data point to the robust estimate of location, see Methods). Outliers are detected using a modification of the box-plot rule. Here, we show that this modification of the box-plot rule offers very high specificity, whilst preserving good sensitivity.

### Simulations

A normal bivariate population of 100 data points [mu(0,0), sigma([1, 0.5; 0.5, 1])] was generated and 10% of outliers added. Outliers came from a similar bivariate population rotated by 90° and shifted along one dimension by 0, 2, 4, or 6 standard deviations (SD) (Figure A1). For each type of outliers (=amount of shift), 1000 Monte-Carlo were performed and the average false positive and true positive rates computed for 11 different methods. We used eight robust methods for which the outlier detection is based on the deviation from the median of projected data: the standard box-plot rule (deviation from the interquartile range), the box-plot rule with Carling’s (2000) adjustment or our own adjustment (as implemented in the skipped correlation), the MAD-median rule (Hall and Welsh, 1985) with or without correction for finite sample size, and adjusted or not, and the S-outlier method (median of absolute deviations – Rousseeuw and Croux, 1993). For comparisons, we also added three non-robust methods for which the outlier detection is based on the deviation from the mean: a simple empirical rule consisting of removing data points located at ±3.29 SD from the mean in at least one marginal distribution, and deviation from the Mahalanobis or bootstrap Mahalanobis distance (with 10,000 resamples, Schwarzkopf et al., 2012), which rely on the bivariate mean(s). The false positive rate corresponds to the number of data points removed from the initial bivariate population; the true positive rate corresponds to the number of data points removed among the 10 added data points. We thus used a conservative definition of outliers as data points originating from a different population. In practice it is however difficult to identify such data points as illustrated in our simulation for 0 shift. In that case, only a subset of observations would appear to be inconsistent with the remainder of the data (Barnett and Lewis, 1994). Overall performance was evaluated via Matthews correlation coefficients, which is the ratio between the difference of contingency table diagonal product [(true positive × true negative) − (false positive × false negative)] and the square root of the products of marginal sums (Bakli et al., 2000).

### Results

Techniques that rely on the mean performed the worst: they showed high specificity because they failed to detect outliers (i.e., they have low sensitivity). In our simulations, the best method only achieved 74.7% [73.6; 75.7] detection for outliers located at 4 SD from the population of interest, and 92.6% [91.99; 93.34] detection for outliers located at 6 SD from the population of interest. For such obvious outliers, robust methods showed 100% or close to 100% detection rates. Among robust methods, the box-plot rule with adjustment as implemented in the skipped correlation function had the highest specificity, i.e., it removed very few data points from the population of interest (Table A1) but at the cost of lower sensitivity than other robust techniques, i.e., it identified fewer true outliers (Table A2). When outliers were close to the population of interest, the box-plot rule with adjustment performed poorly, but as outliers were farther away from the population of interest, it outperformed techniques that had higher false positive rates (see Matthews correlation coefficients, Figure A1).

**Table A1:** **False positive rate (average number of data points detected as outliers from the normal data) and 95% percentile CIs for the different outlier detection methods**.

	Distance to the center = 0	Distance to the center = 2	Distance to the center = 4	Distance to the center = 6
Marginal means	0.10 [0.07, 0.13]	0.032 [0.01, 0.05]	0.01 [0.006, 0.028]	0.02 [0.007, 0.03]
Mahalanobis distance	2.96 [2.85, 3.08]	1.80 [1.71, 1.90]	1.0070 [0.9228, 1.091]	0.86 [0.79, 0.93]
Bootstrapped Mahalanobis distance	3.44 [3.31, 3.56]	2.20 [2.09, 2.31]	1.26 [1.17, 1.35]	1.07 [0.9942, 1.15]
Box-plot	6.40 [6.18, 6.63]	4.94 [4.73, 5.15]	3.9610 [3.78, 4.13]	3.67 [3.50, 3.85]
Box-plot with Carling’s adjustment	4.56 [4.36, 4.76]	3.45 [3.2873, 3.61]	2.7570 [2.6, 2.91]	2.56 [2.42, 2.69]
Box-plot adjusted for bivariate data	1.91 [1.78, 2.04]	1.28 [1.17, 1.39]	0.9570 [0.87, 1.04]	0.96 [0.87, 1.04]
MAD-median rule	15.41 [15.11, 15.71]	13.23 [12.94, 13.52]	11.7580 [11.47, 12.03]	11.3 [11.01, 11.58]
MAD-median rule adjusted for bivariate data	9.23 [8.97, 9.49]	7.60 [7.35, 7.85]	6.5610 [6.33, 6.78]	6.29 [6.07, 6.5]
MAD-median rule for finite samples	15.15 [14.85, 15.45]	13.01 [2.71, 13.32]	11.5460 [11.27, 11.82]	11.11 [10.82, 11.40]
MAD-median rule for finite samples adjusted for bivariate data	9.04 [8.78, 9.30]	7.45 [7.20, 7.71]	6.4070 [6.18, 6.62]	6.13 [5.92, 6.35]
S-outliers	15.63 [15.34, 15.91]	13.61 [13.31, 13.90]	12.6660 [12.41, 12.92]	12.84 [12.55, 13.13]

**Table A2:** **True positive rate (average number of data points detected as outliers from the outlier data) and 95% percentile CIs for the different outlier detection methods**.

	Distance to the center = 0	Distance to the center = 2	Distance to the center = 4	Distance to the center = 6
Marginal means	5.67 [5, 6.33]	6.91 [6.24, 7.57]	11.8900 [11.04, 12.73]	15.9400 [15.01, 16.86]
Mahalanobis distance	28.77 [27.72, 29.81]	43.96 [42.84, 45.07]	69.6500 [68.61, 70.68]	88.0900 [87.31, 88.86]
Bootstrapped Mahalanobis distance	30.55 [29.53, 31.56]	46.74 [45.55, 47.92]	74.7300 [73.68, 75.77]	92.6700 [91.99, 93.34]
Box-plot	38.65 [37.42, 39.87]	61.2400 [59.91, 62.56]	95.7900 [95.20, 96.37]	99.9800 [99.98, 100]
Box-plot with Carling’s adjustment	35.64 [34.45, 36.82]	56.8100 [55.47, 58.14]	94.0600 [93.37, 94.74]	99.96 [99.96, 100]
Box-plot adjusted for bivariate data	28.56 [27.33, 29.78]	46.5900 [45.19, 47.98]	87.5000 [86.55, 88.44]	99.8500 [99.69, 100]
MAD-median rule	49.5 [48.12, 50.87]	74.7800 [73.61, 75.94]	98.6800 [98.32, 99.03]	100
MAD-median rule adjusted for bivariate data	42.9700 [41.58, 44.35]	66.8700 [65.6, 68.13]	97.4800 [97, 97.95]	100
MAD-median rule for finite samples	49.19 [47.8, 50.57]	74.5800 [73.34, 75.81]	98.6200 [98.25, 98.98]	100
MAD-median rule for finite samples adjusted for bivariate data	42.64 [41.3, 43.97]	66.5100 [65.22, 67.79]	97.3400 [96.8605, 97.81]	100
S-outliers	49.9 [48.58, 51.21]	75.1400 [73.89, 76.38]	98.8000 [98.50, 99.09]	100
